# The role of vitamin D3 in follicle development

**DOI:** 10.1186/s13048-024-01454-9

**Published:** 2024-07-17

**Authors:** Mingxia Li, Shuhui Hu, Jiaxiang Sun, Ying Zhang

**Affiliations:** 1 Obstetrics and Gynecology Hospital, Fudan University, Fangxie Road 419, Shanghai, Huangpu 200011 China; 2 The Shanghai Key Laboratory of Female Reproductive Endocrine-Related Diseases, Shanghai, 200011 China

**Keywords:** Vitamin D3, Follicle development, Granule cells

## Abstract

Vitamin D3 plays a crucial role in female reproduction. As research progresses, the mechanisms of action of vitamin D3 on follicular development have been widely discussed. Firstly, key enzymes involved in the synthesis and metabolism of vitamin D3 have been discovered in the ovary, suggesting that vitamin D3 can be synthesized and metabolized locally within the ovary. Additionally, the detection of vitamin D3 receptors (VDR) in follicles suggests that vitamin D3 may exert its effects by binding specifically to these receptors during follicular development. Further research indicates that vitamin D3 promotes follicular growth by enhancing the development of granulosa cells (GCs) and oocytes. Currently, the mechanism of action of vitamin D3 in follicular development is becoming increasingly clear. Vitamin D3 promotes oocyte development by regulating molecules involved in meiotic arrest in oocytes. It also enhances granulosa cell proliferation by stimulating steroid hormone synthesis and cell cycle regulation. Additionally, vitamin D3 exerts anti-inflammatory effects by reducing oxidative stress and advanced glycation end-products (AGEs), mitigating the detrimental effects of inflammation on follicular development. These functions of vitamin D3 have clinical applications, such as in treating polycystic ovary syndrome (PCOS), improving female fertility, and enhancing outcomes in in vitro fertilization (IVF). This review summarizes the research progress on the role and mechanisms of vitamin D3 in follicular development and briefly summarizes its clinical applications.

**Supplementary Information:**

The online version contains supplementary material available at 10.1186/s13048-024-01454-9.

## Introduction

In the human body, vitamin D3 is the primary form of vitamin D, primarily synthesized from 7-dehydrocholesterol in the skin upon exposure to ultraviolet radiation, exerting its biological effects through binding to Vitamin D receptors (VDRs). The classical functions of vitamin D3 include regulating the homeostasis of calcium and phosphorus in the blood circulation [1], and support the growth and maintenance of the skeleton [2]. Moreover, vitamin D3 plays a regulatory role in cell differentiation [3], inflammation [4] and apoptosis [5, 6]. In recent years, an increasing number of people have come to realize the crucial role of maintaining normal physiological levels of Vitamin D3 in sustaining normal ovarian function and female reproductive physiology [7]. Vitamin D deficiency has been linked to adverse maternal-fetal outcomes and specific gynecological conditions that affect fertility, such as infertility [8], polycystic ovary syndrome (PCOS) [9], and endometriosis [10]. Moreover, studies have shown that supplementing with vitamin D3 can improve insulin resistance [11], lower androgen levels [12], and increase pregnancy rates [13] in patients with PCOS. Furthermore, adding vitamin D3 can also improve the success rates of in vitro fertilization (IVF) for patients experiencing infertility [14].

Follicles are the fundamental functional units of the ovary, comprising follicle cells and developing oocytes. Based on the developmental stage, follicles can be classified as primordial, primary, secondary, antral, and mature follicles. As the follicle develops, the pre-granulosa cells within the primordial follicle gradually mature, initiating the expression of follicle-stimulating hormone receptors, estrogen receptors, and androgen receptors, marking the follicle’s transition into the hormone-dependent stage of development. The formation of primordial follicles and their subsequent development are crucial processes in ovarian biology, directly influencing the number of available oocytes [15]. Understanding the mechanism of action of vitamin D in the process of follicular development holds significant value for its clinical application. The following sections will combine recent research to review the role of vitamin D in regulating follicular development and maturation, as well as its potential signaling pathways.

### Vitamin D3 metabolism and VDR expression in the ovary

Vitamin D3 is typically converted into its biologically active form, 1α,25-dihydroxyvitamin D3 (1α,25(OH)2D3, calcitriol), primarily by the hepatic 25-hydroxylase and the renal 1-α-hydroxylase [16], and subsequently metabolized by 24-hydroxylase [17]. Studies have shown that, in addition to its synthesis in classical organs, vitamin D3 can also be locally synthesized in the ovary. 25-hydroxylase and 1-α-hydroxylase, the key enzymes in the synthesis of calcitriol, which are respectively encoded by CYP2R1 and CYP27B1 genes. Their mRNA was expressed in human ovary and in developing follicles of non-human primate (rhesus monkey) [17, 18].

In a study of in vitro cultured rhesus monkey follicles, Xu et al. observed that the mRNA levels of CYP2R1 were higher in small antral follicles compared to preantral follicles (*P* < 0.05), while the mRNA levels of CYP27B1 showed no significant variation [17]. In contrast, in a study on pig follicles, Grzesiak found that the mRNA and protein levels of CYP27B1 were higher in medium-sized antral follicles compared to small antral follicles (mRNA level: 1.55-fold, *P* = 0.042; protein level: 2.49-fold, *P* = 0.005). Additionally, the mRNA levels of CYP24A1 were also higher in medium-sized antral follicles (1.96-fold; *P* = 0.042), while there was no statistically significant difference in its protein levels across follicular stages [19]. These differences may be attributed to racial disparities and variations in the developmental stages of the analyzed follicles. These studies collectively indicate an increase in the expression of vitamin D3 synthesizing enzymes as follicles mature. Furthermore, supplementation of vitamin D3 in the culture medium resulted in a significant increase in the concentration of CYP2R1 mRNA in preantral follicles after two weeks, suggesting that vitamin D3 supplementation can positively feedback to promote the synthesis of vitamin D3 in preantral follicles.

However, the realization of vitamin D3 action also depends on the distribution of its receptor (vitamin D receptor, VDR). In the presence of ligands, VDR forms heterodimeric complexes with the retinoic X receptor (RXR) to create VDR/RXR heterodimeric complexes [20]. This complex binds to vitamin D response elements in the promoter regions of target genes, regulating gene expression through signal cascades, thereby achieving biological effects [21].

In 1983, Dokoh et al. found the expression of VDR in the ovaries of mammals and birds [22]. Subsequently, immunofluorescent staining of VDR was also observed in oocytes of primordial follicles in fish [23], goats [24], and rhesus monkeys [17]. In contrast, in primary and secondary follicles of rats and goats, VDR signaling is predominantly expressed in granulosa cells (GCs) and theca cells (TCs) [24, 25]. In studies on ovarian tissue of rhesus monkeys, Xu used immunohistochemical staining to assess the protein expression levels of VDR [17]. The results of the study showed that VDR was expressed at all stages of oocyte development. Although VDR expression was also present in pregranulosa cells of primordial follicles, it was less pronounced. Additionally, only trace amounts of VDR staining were observed in granulosa cells in primary follicles. However, as the follicles progressed through the stages of secondary, small antral, and large antral follicles, the positive staining of granulosa cells became increasingly prominent. This suggests that VDR expression in follicles is stage-dependent and gradually increases as follicles develop and mature.

Yao et al. also confirmed that the mRNA and protein expression levels of VDR in goat granulosa cells significantly increased with the increase in follicle diameter (*P* < 0.05) [24]. These findings collectively indicate that the expression of VDR in follicles is stage-dependent and gradually increases with follicular development and maturation. Xu et al. also discovered that for follicles cultured in vitro, VDR mRNA levels were lower compared to those developing in vivo, but upon addition of vitamin D3, they were restored to in vivo levels [17]. Grzesiak et al. observed the highest expression levels of VDR mRNA in small antral follicles in pig antral follicles cultured in vitro, demonstrating a gradual decrease in VDR mRNA expression during the development of follicles cultured in vitro [19]. This variation may be attributed to the depletion of vitamin D3 in the in vitro culture. Studies have indicated that the concentration of vitamin D3 can dynamically regulate the number of VDR/RXR heterodimeric complexes on VDR binding sites, thereby influencing VDR expression [26, 27]. However, the specific molecular mechanism underlying this process remains to be elucidated.

### Vitamin D3 and follicular development

Previous studies suggest that vitamin D3 may be involved in follicular development. In a control experiment using CYP27B1 knockout mice, Dicken et al. found that a diet deficient in vitamin D3 led to delayed ovarian development and prolonged estrous cycles in mice [7]. However, when vitamin D3 was added to the diet, the estrous cycles returned to normal. Xu et al. investigated the effects of vitamin D3 on follicular development using a three-dimensional cultured rhesus monkey follicle model [28]. Their results showed that after 5 weeks of in vitro culture, both low-dose (25 pg/ml) and high-dose (100 pg/ml) vitamin D3 treatments led to an increase in the diameter of antral follicle oocytes compared to the control group (*P* < 0.05). Additionally, the high dose of vitamin D3 also increased the proportion of growing follicles, indicating its promotion of antral follicle growth and direct stimulation of follicular development. This article, in conjunction with the latest research advancements, provides a review of the possible mechanisms involved.

**Fig. 1:**
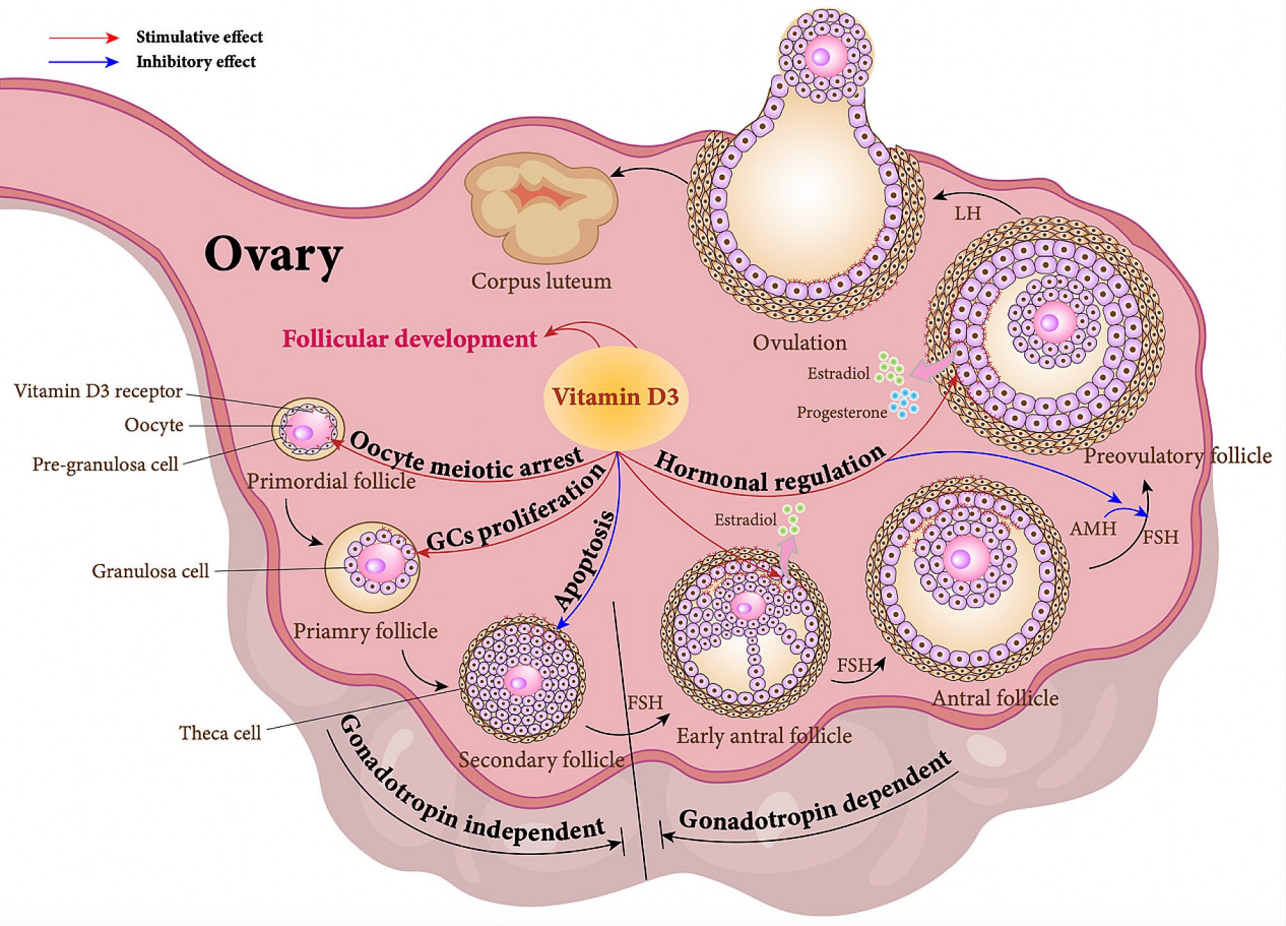
The role of vitamin D3 in follicular development. According to the developmental stages of follicles, follicles can be classified into primordial follicles, primary follicles, secondary follicles, antral follicles, and mature follicles. During the transition from secondary to antral follicles, hormone receptors begin to express, entering the stage of gonadotropin dependence. Vitamin D3 promotes follicular development by facilitating oocyte meiosis, stimulating granulosa cell proliferation, enhancing the secretion of estrogen and progesterone in follicular cells, inhibiting the inhibitory effect of AMH on FSH, reducing cellular apoptosis. (AMH: anti-Müllerian hormone; FSH: follicle-stimulating hormone. Red arrows represent facilitation and blue arrows represent inhibition.)

### Oocyte

The cell cycle of growing oocytes halts at the first meiotic prophase and resumes only a few hours before ovulation. During this period, oocytes accumulate numerous transcripts and proteins to support early embryo development [29]. The process is achieved through the C-type natriuretic peptide (NPPC) pathway. NPPC, secreted by the mural granulosa cells (MGC), binds to its specific receptor NPR2, initiating the production of cyclic guanosine monophosphate (cGMP). Subsequently, cGMP enters the oocyte via gap junctions, leading to elevated levels of cyclic adenosine monophosphate (cAMP) and the maintenance of oocyte meiotic arrest [30] (Fig. 1). Transforming growth factor-beta (TGF-β), a growth factor family closely associated with Smads, upon binding to its receptor TGFR, activates Smad2/3. Activated Smads2/3 form heteromeric complexes with Smad4 and translocate to the nucleus [31], where they bind to the NPPC promoter region, significantly increasing NPPC levels in cultured MGCs(Fig. 1).

Corduk et al. conducted a well-designed randomized controlled trial with 24 mice. The experimental group received injections of vitamin D3 (0.05 µg/kg, once every other day) for 8 weeks [32]. Compared to the control group, adult rat ovarian TGF-β1 immunostaining was negative after vitamin D3 supplementation, suggesting that vitamin D3 may promote oocyte development by restoring the meiotic status of oocytes through reducing TGF-β1 levels. However, their study did not find a significant decrease in TGF-β1 expression in the ovaries of newborn rats, possibly due to the immature development of VDR in newborn rat ovaries. In addition, Han et al. observed that vitamin D3 supplementation reduced the expression of TGF-β1 and phosphorylated Smad-2/3 in lung tissue using primary lung cells from TGF-β1 transgenic mice [33]. Similarly, Wang et al. demonstrated in their study that vitamin D3 ameliorated renal fibrosis in rats with chronic kidney disease (CKD) by upregulating VDR and inhibiting the TGF-β1/Smad3 signaling pathway [34]. These findings suggest that vitamin D3 can inhibit the TGF-β1/Smad3 signaling pathways, thereby potentially reducing NPPC levels, overcoming oocyte meiotic arrest, and promoting follicular development.

After binding of NPPC to its receptor, the resulting cGMP needs to pass through gap junction proteins to enter the oocyte, thereby maintaining the meiotic arrest of the oocyte. Connexin 43 (Cx43) and connexin 37 (Cx37) are two crucial gap junction proteins in follicular development, playing a key role in sustaining meiotic arrest. Richard et al. utilized connexin mimetic peptides (CMPs) to disrupt gap junctional heteromers [35], and they found that adding Cx43 CMP to the culture medium significantly increased the rate of oocyte meiotic resumption in cumulus-oocyte complexes (COCs). On the other hand, Cx37 CMP had no effect when added to the culture medium, but when injected in trace amounts into the perioocyte space near the oocyte surface, it could release the oocyte blockade. This is consistent with previous reports that Cx43 is situated in the cumulus cells, while Cx37 is found on the surface of the oocyte [36]. Additionally, Ackert et al. found that in mice with transplanted ovaries lacking Cx43, the cytoplasm of oocytes and granulosa cells became vacuolated, resulting in delayed oocyte growth and failure to complete final meiotic divisions and fertilization [37]. Furthermore, research has shown that compared to the control group, nuclear maturation and embryo development significantly increased after in vitro fertilization (IVF) of bovine oocytes treated with 25 µg/mL Cx37 [38]. All these findings suggest that Cx43 and Cx37 are critical for the maturation of oocyte development.

Previous studies have indicated that vitamin D3 can increase the expression of Cx43 in rat hepatic stellate cells, enhancing intercellular communication [39]. Based on this, Lee et al. found that in primary cultured rat granulosa cells, vitamin D3 (0.1µM) can reverse the inhibitory effect of high-dose testosterone (1 µg/mL) on Cx43 expression in granulosa cells, thereby promoting follicular development [40].

### GC proliferation

During follicular development, GC can provide the necessary physical support and microenvironment for oocyte development. Wojtusik et al. found in vitro that both low (10nM) and high (100nM) doses of vitamin D3 can promote proliferation of GC in hen follicles [41]. Subsequently, Yao et al. confirmed that vitamin D3 promotes the proliferation of luteinized goat GC in a dose-dependent manner [24]. The regulation of GC proliferation by vitamin D3 can be achieved through several mechanisms.

Cell cycle regulation is an essential component of cellular growth and development, governed by cell cycle proteins, cyclin-dependent kinases (CDKs), and cyclin-dependent kinase inhibitors (CKIs). Yao et al. confirmed in vitro that the use of vitamin D3 alone (10nM) promotes proliferation of GC in 2–5 mm diameter follicles of goats (*P* < 0.05) [24]. They also observed that following treatment with vitamin D3 (10nM) for 48 h, the mRNA levels of Cyclin-dependent kinase 4 (CDK4) and Cyclin D1 significantly increased in GC, while the mRNA levels of CKIs such as P21 significantly decreased. Additionally, flow cytometry results showed a significant increase in the proportion of S-phase cells and a decrease in the proportion of G0/G1-phase cells in GC treated with vitamin D3. Previous studies have demonstrated that activation of the Cyclin D1-CDK4 complex is necessary for cells to reach the G1/S restriction point [42]. Subsequently, they observed opposite results in GC with knocked down VDR [43], further confirming that vitamin D3 upregulates the expression of CDK4 and Cyclin D1 while inhibiting P21 expression, thereby activating pRb, releasing the transcription factor E2F, and inducing cell cycle progression from G0/G1 phase to S phase. These findings suggest that vitamin D3 indirectly affects follicular development by regulating the cell cycle to promote GC proliferation.

However, when exposed to a high dose of vitamin D3 (100 nM) for 48 h, contrary effects were observed, consistent with previous findings in tumor cells [44]. Meanwhile, Wojtusik et al. found that adding vitamin D3 (100 nM) to in vitro cultures of hen follicles for 24 h promoted GC proliferation [41], which could be attributed to the shorter duration of vitamin D3 treatment and species differences. Additionally, interactions between GCs and other cells within the cultured follicles may also influence experimental outcomes. Therefore, further experiments are needed to elucidate the effects of high-dose vitamin D3 on follicular development and granulosa cell proliferation, as well as its underlying mechanisms.

### Hormonal regulation

Follicular growth and development are regulated by various hormones and growth factors, such as follicle-stimulating hormone (FSH), luteinizing hormone (LH), epidermal growth factor (EGF), insulin-like growth factor (IGF), and inhibitory substances [namely anti-Müllerian hormone (AMH)] [45]. Vitamin D3 can modulate multiple hormones required for follicular growth, thereby influencing follicular development.

### AMH

Chu et al. found in their study on 351 healthy reproductive-age women that both total and free serum vitamin D3 levels were negatively correlated with serum AMH levels [46]. AMH is a member of the TGF-β superfamily regulated by the Smads gene, first expressed in GCs of preantral and early antral follicles [47]. Peak expression of AMH gene and its protein occurs in human follicles with a diameter of 8 mm, followed by a sharp decline with increasing follicular diameter [48]. AMH receptor II (AMHR-II) is expressed in the granulosa cells and follicular membrane cells of preantral and small antral follicles, but in large antral or pre-ovulatory follicles, they are only expressed in the follicular membrane cells [49]. AMH is one of the major regulatory factors in the early stages of folliculogenesis [50]. It suppresses the assembly rate of primordial follicles and inhibits the development of primordial follicles by inhibiting apoptosis of oocytes. However, during the hormone-dependent stage of follicular development, AMH reduces the responsiveness of growing follicles to FSH by inhibiting the expression of FSH receptors, thereby regulating the recruitment of primordial follicles and the selection of dominant follicles [51].

Merhi et al. collected follicular fluid from 54 IVF patients and found a negative correlation between vitamin D3 levels in follicular fluid and mRNA levels of AMH and AMHR-II in cumulus GCs of small antral follicles (*P* < 0.05), while no significant difference was observed in mural GCs [52]. Subsequently, they treated isolated cumulus GCs with vitamin D3 (50nM) for 24 h and found a 36% reduction in AMHR-II levels in the treated group compared to the control group (*P* < 0.05), but no statistically significant difference in AMH protein levels. Additionally, compared to using recombinant AMH alone (50ng/mL), the combination of vitamin D3 and recombinant AMH led to a more significant decrease in nuclear phosphorylation of Smad 1/5/8 in granulosa cells after treatment (*P* < 0.0001). This suggests that vitamin D3 can inhibit the action of AMH by reducing the phosphorylation sites and nuclear localization of the Smads family (Smad 1/5/8) and suppressing the expression of AMHR-II to counteract the inhibitory effect of AMH on GCs, promoting follicular maturation. However, Malloy et al. found in human prostate cancer cells that there is a sequence in the promoter of the AMH gene similar to the vitamin D response element and demonstrated that vitamin D3 can upregulate the expression of the AMH gene through functional VDRE bound by VDR [53]. This may be due to differences in cell types and species. A recent systematic review and meta-analysis showed that supplementation with vitamin D3 affects serum AMH levels in relation to ovulation status in patients. After vitamin D3 supplementation, serum AMH levels significantly decreased in anovulatory PCOS patients[Standardized Mean Difference (SMD) -0.53, 95% CI -0.91 to -0.15, *p* < 0.007], while they significantly increased in ovulatory non-PCOS women (SMD 0.49, 95% CI 0.17 to 0.80, *p* = 0.003) [54]. This finding indicates that the regulation of AMH by vitamin D3 may be bidirectional. Therefore, further clinical studies controlling for factors such as ovulation status, season, diet, and patient BMI are needed to further explore the complex regulatory effects of vitamin D3 on AMH. Additionally, further investigation is required to determine whether the dosage of vitamin D supplementation should be adjusted based on the levels of vitamin D in patients’ bodies.

**Fig. 2:**
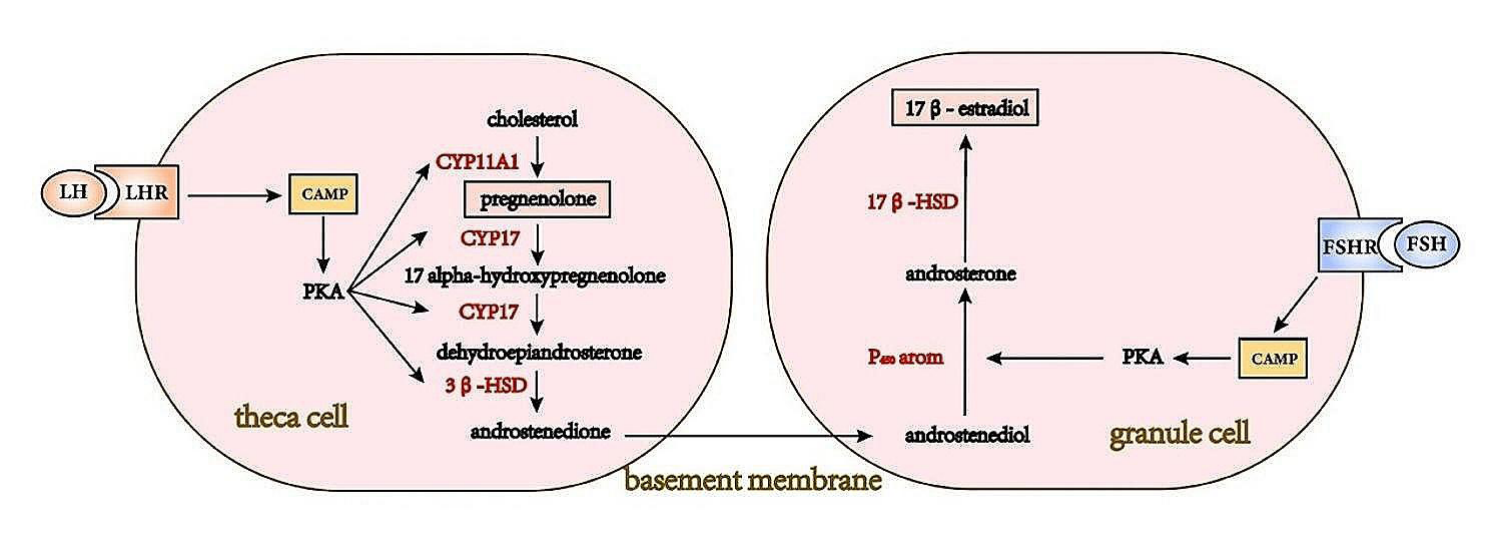
Pathways for the synthesis of estrogen and progesterone. Estrogen and progesterone synthesis occurs within the follicular cells. CYP11A1, CYP17, 3β-HSD, 17β-HSD and P450arom are genes related to steroidogenic enzymes, collectively regulating the synthesis of estrogen and progesterone. (P450arom encodes aromatase, which is the rate-limiting enzyme in estrogen synthesis.)

### Estradiol

Harmon et al. found in a retrospective study of 89 reproductive-age women that those with low serum vitamin D levels (< 30 ng/ml) had lower average concentrations of estradiol (E2) throughout the menstrual cycle [55]. Furthermore, Parikh et al. discovered in vitro cultured human ovarian tissue that the addition of vitamin D3 alone (50nM) increased estradiol synthesis by 9% (*p* < 0.02), while simultaneous supplementation with vitamin D3 and insulin increased estradiol synthesis by 60% (*p* < 0.005), indicating a synergistic effect of vitamin D3 and insulin in stimulating estradiol production in human ovaries [56]. The synthesis of E2 is essential for normal follicular development and ovulation, and this process is completed in GCs (Fig. 2). Supplementing vitamin D3 increased the conversion rate of androstenedione to estrone in human skin fibroblasts [57], and it may exert a similar effect in granulosa cells. Subsequently, Hong et al. further investigated the mechanism of action of vitamin D3 in vitro cultured pig granulosa cells [58]. They found that after 24 h of culture with vitamin D3 (100nM), the levels of estrogen synthesis-related genes (such as CYP17A1, HSD17B1, and CYP19A1 mRNA) in granulosa cells were significantly upregulated compared to the control group, while the upregulation of CYP17A1 and CYP19A1 protein levels was not significant (Fig. 2). This suggests that vitamin D3 can increase the expression of genes involved in estrogen biosynthesis, while the changes in protein levels may be related to the concentration of vitamin D and the duration of treatment. Recently, Cheng et al. fed laying hens with different concentrations of vitamin D3 (10 and 100 µg/kg VitD3) for 4 weeks and found that the levels of serum E2 and P4 in the treatment group significantly increased [59]. Transcriptome sequencing of ovarian tissue showed that vitamin D3 supplementation altered the gene expression of ovarian steroidogenesis pathways. Compared to the control group, the expression of the estrogen synthase gene HSD17B1 mRNA was higher in the low-dose vitamin D3 group, while it was lower in the high-dose vitamin D3 group. This indicates that adequate vitamin D3 promotes E2 production in granulosa cells by increasing the expression of the estrogen synthase gene (HSD17B1). Estrogen synthase gene converts testosterone to 17β-estradiol in GCs, aiding in follicle maturation. However, excessive levels of testosterone may inhibit its activity. Lee et al. induced GCs from primary cultures of rats with high doses of testosterone to simulate the high androgen status in patients with PCOS [40]. The study found that 0.1µM vitamin D3 significantly improved the inhibitory effect of high-dose testosterone (1 µg/mL) on aromatase levels, possibly by increasing the phosphorylation level of aromatase tyrosine.

### Progesterone

**Fig. 3:**
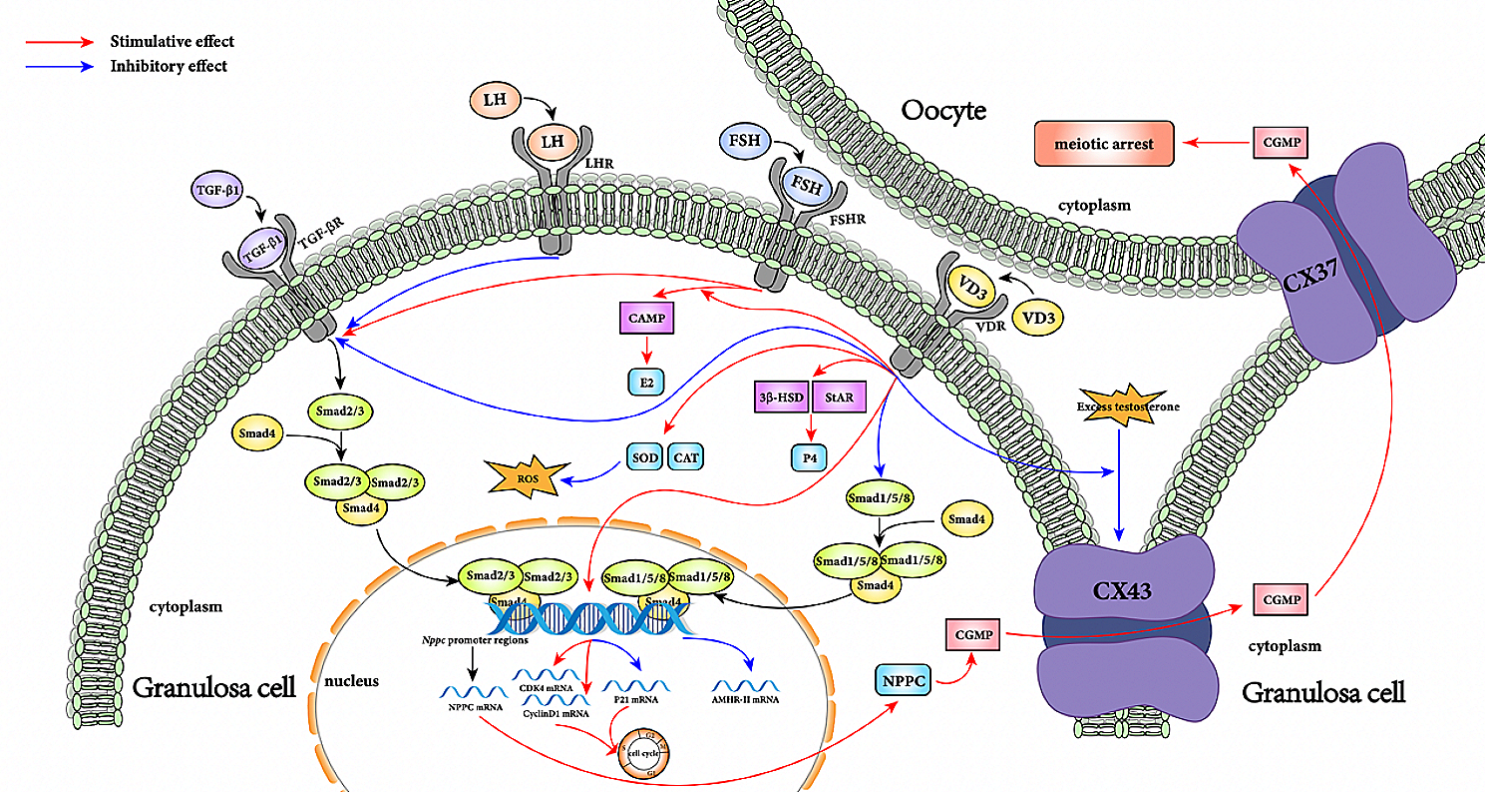
The molecular mechanism by which vitamin D3 promotes follicular development. TGF-β1 binds to its receptor, activating Smad2/3, which then forms a heteromeric complex with Smad4 and translocates into the nucleus. There, the complex binds to the NPPC promoter region, promoting NPPC synthesis. NPPC, in turn, stimulates cGMP synthesis, which enters oocytes through CX43 and CX37, maintaining oocyte meiotic arrest. Vitamin D3 promotes the resumption of oocyte meiosis by inhibiting the TGF-β1 pathway. Vitamin D3 can reverse the inhibitory effect of excess testosterone on CX43. LH downregulates the TGF-β1 pathway, while FSH upregulates it. Vitamin D3 promotes granulosa cell proliferation by upregulating the expression of CDK4 and CyclinD1 mRNA and inhibiting P21 mRNA expression. It also enhances granulosa cell secretion of E2 and P4 by promoting the expression of steroidogenic enzymes, thereby promoting follicular development. Furthermore, vitamin D3 reduces the synthesis of AMHR-II mRNA by inhibiting SMAD 1/5/8 phosphorylation. By upregulating SOD and CAT levels, vitamin D3 reduces ROS generation, exerting anti-inflammatory effects and mitigating the adverse effects of ROS on follicular development. (TGF-β1: transforming growth factor-β; NPPC: natriuretic peptide type C Cx43: connexin 43; CDK4:cyclin-dependent kinases 4; E2: estradiol; P4: progesterone SOD: Superoxide dismutase; CAT: catalase; ROS: reactive oxygen species. Red arrows represent facilitation and blue arrows represent inhibition.)

Serum estrogen and progesterone levels were reduced in mice lacking 25-hydroxyvitamin D1α-hydroxylase, suggesting that vitamin D3 deficiency may lead to a decrease in progesterone (P4) [60]. P4 is produced by granulosa cells and follicular membrane cells of follicles, supporting follicular development. The synthesis of P4 is a complex process regulated by steroidogenic acute regulatory protein (StAR) and 3β-hydroxysteroid dehydrogenase(3β-HSD) [61](Fig. 2). Yao et al. also found in their study on goat granulosa cells that treatment with vitamin D3 (10nM) or FSH (10 ng/ml) alone for 48 h significantly increased P4 production (*P* < 0.05), but vitamin D3 did not enhance the promotive effect of FSH on P4 [24]. Furthermore, they found that compared to the control group, the expression of 3β-HSD and StAR mRNA in GCs of the vitamin D3-treated group was significantly upregulated, suggesting that vitamin D3 promotes P4 synthesis by enhancing the expression of steroidogenic enzyme-related genes, thereby promoting follicular development (Fig. 3). This is consistent with previous findings by Merhi et al., who demonstrated that supplementation with vitamin D3 (50 nm) increased P4 synthesis and release in cumulus GCs, resulting in increased levels of 3β-HSD mRNA and 3β-HSD enzyme activity [52]. This suggests that the promotion of P4 synthesis by vitamin D3 may involve a positive feedback process that requires further experimental validation.

Recently, Cheng et al. found that serum P4 levels significantly increased after supplementing vitamin D3 in the diet of laying hens, while serum total cholesterol levels decreased [59]. Subsequently, they found that after 24 h of culturing chicken follicles in vitro with vitamin D3 (10nM and 100nM), the levels of P4 in granulosa cells and follicular membrane cells significantly increased. Further PCR results confirmed that 10 nm vitamin D3 significantly increased the level of CYP11A1 mRNA. The enzyme encoded by CYP11A1 can convert cholesterol into P4, promoting the synthesis of P4. These findings indicate that vitamin D can promote follicular development by increasing the level of CYP11A1 mRNA, facilitating the conversion of serum cholesterol for progesterone synthesis.

### Anti-inflammatory effects

The regulation of follicular development by vitamin D3 includes not only increasing support from various molecules and hormones but also reducing some adverse factors in follicular development, such as clearing reactive oxygen species (ROS) and advanced glycation end products (AGEs).

Oxidative stress is one of the initiating factors of oocyte aging, which can lead to abnormal closure of sinusoidal follicles and meiotic abnormalities in oocytes, thus affecting follicular development. In a randomized controlled trial conducted by Razavi et al. on 60 patients with PCOS, they found that supplementation with vitamin D-K-calcium significantly increased the plasma total antioxidant capacity (TAC) and significantly reduced the concentration of plasma malondialdehyde (MDA). However, this study did not analyze the individual effects of supplementation with vitamin D3 or the concentration of vitamin D3 on patients with PCOS. Subsequently, Yao et al. found that the use of vitamin D3 alone (10 nM) significantly reduced the content of ROS in goat GCs and upregulated the mRNA and protein levels of antioxidant-related enzyme genes [superoxide dismutase 2 (SOD2) and catalase (CAT)], indicating that vitamin D3 reduces ROS levels and promotes GC proliferation by promoting the expression of SOD and CAT [24]. Recently, Jiang et al. induced granulosa-like tumor cells with high androgen levels to simulate the changes in GCs in patients with PCOS. They found that vitamin D3 (100 nM) effectively inhibited the increase in MDA, ROS, and lipid peroxidation levels induced by high androgens, reducing oxidative stress levels and enhancing the vitality of GCs. This provides new insights into the potential application of vitamin D3 in the treatment of PCOS.

Advanced glycation end products(AGEs) are cytotoxic metabolic byproducts that bind to the receptor for advanced glycation end products (RAGE) on GCs and TCs, leading to cellular dysfunction and abnormal follicle growth [62]. Chatzigeorgiou et al. found that in rats fed with high-in-AGE (H-AGE) diet, E2 and P4 levels decreased while testosterone levels increased, and the expression of RAGE decreased by 30%, indicating that AGEs can adversely affect follicle development by interfering with steroid hormone synthesis [63]. Furthermore, research suggests that AGEs can disrupt intracellular insulin signaling and glucose transport in granulosa cells, resulting in abnormal follicle development [64]. Soluble RAGE (sRAGE) competes with free AGEs by binding to them and exerting inhibitory effects [65]. Vitamin D3 significantly increases the levels of sRAGE in the ovaries, thereby reducing the negative impact of accumulated AGEs in the GCs and TCs on follicle development [65]. Guo et al. found a close association between vitamin D3 and the AGE-RAGE axis, showing inhibitory effects of vitamin D3 on inflammation induced by AGEs in an in vitro blood-brain barrier model [66]. Additionally, in vitro studies on human GCs indicate that vitamin D3 attenuates the effects of AGEs on the expression of genes related to steroidogenesis in luteinized GCs, such as inhibiting the increase in mRNA levels of CYP11A1, StAR, and CYP17A1 induced by AGEs [67, 68]. Other studies have shown that AGEs enhances the phosphorylation of SMAD 1/5/8 induced by recombinant AMH, while adding VD3 to AGEs reverses this phenomenon and suppresses the elevated levels of AMHR-II mRNA induced by AGEs [67]. These studies suggest that vitamin D3 can maintain normal follicle development by reducing the expression of AGEs. Their research subjects were granulosa cells luteinized after ovulation induction in IVF patients, neglecting the influence of vitamin D3 on non-luteinized granulosa cells, which requires further investigation.

### Clinical applications

Vitamin D3 plays an important role in follicular development, which is closely related to female reproductive system diseases and fertility.

In a double-blind, multicenter trial involving 750 women with PCOS, Butts et al. found that compared to women with normal levels of vitamin D3, those with a deficiency in vitamin D3 (vitamin D3 < 20 ng/mL) were less likely to ovulate and had a lower live birth rate. Fang et al. conducted a meta-analysis of nine randomized controlled trials comparing the efficacy of vitamin D3 supplementation with placebo or metformin. The analysis revealed that vitamin D supplementation significantly promoted follicular development, leading to an increase in the number of dominant follicles (OR = 2.34; 95% CI: 1.39 ~ 3.92), ultimately improving pregnancy outcomes. However, in a single-center, double-blind, randomized placebo-controlled trial involving 123 PCOS patients, supplementation with vitamin D3 had no significant effect on metabolic and endocrine parameters [69]. These differences may be due to variations in the dosage of vitamin D3 supplementation and population characteristics. More large-scale, high-quality, long-term follow-up randomized controlled studies are needed to explore the threshold effects of vitamin D supplementation on hormones, metabolism, and reproductive outcomes in PCOS patients, as well as to investigate the effects of factors such as dosage, administration method, and timing of vitamin D3 supplementation on efficacy.

Halloran et al. found that compared to the control group, the fertility of the mice with vitamin D3 deficiency declined 75% and the number of live births of them also reduced by 30% [70]. The mice lack of vitamin D3 adversely affect the fertility of the offspring, which is manifested as less ovulation, prolonged and irregular estrous cycle when its grow up. In addition, an inverse association between serum vitamin D3 levels and a marker of ovarian reserve, AMH, has also been reported in several studies [46, 67]. Previous research has demonstrated that vitamin D3 has the potential to directly enhance progesterone secretion in ovarian granulosa cells in vitro [52]. This implies that vitamin D3 may play a role in promoting progesterone secretion in ovarian granulosa cells during early pregnancy, thereby supporting embryo implantation, reducing the probability of miscarriage, and enhancing fertility. This provides new insights and directions for enhancing fertility treatments.

Vitamin D3 levels also play a role in fertility and affect the outcomes of in vitro fertilization (IVF). A prospective study of 80 infertile women undergoing IVF/ICSI showed that vitamin D3 independently increased implantation rates and IVF outcomes without affecting oocyte quantity and quality [71]. Another prospective cohort study of 84 women undergoing IVF for infertility found that compared to women with low initial vitamin D levels (104.3 ± 21 nmol/L), those with high initial vitamin D levels (267.8 ± 66.4 nmol/L) had a fourfold higher IVF success rate (*p* < 0.01). Furthermore, multivariate logistic regression analysis confirmed that the level of 25-hydroxyvitamin D in follicular fluid serves as an independent predictor of a successful IVF cycle [72]. Studies have suggested that vitamin D3 not only promotes follicular development but also influences fertilization processes [73] and embryo implantation [74, 75], thereby affecting IVF outcomes. However, further empirical evidence is needed to establish the exact role and mechanisms of action of vitamin D3 in IVF outcomes.

## Conclusion

It has been reported that vitamin D3 deficiency is becoming more and more common in women [76]. However, vitamin D3 deficiency has extensive adverse effects on the female reproductive system, such as PCOS, decreased fertility, and poor outcomes in assisted reproductive technologies. Therefore, vitamin D3 supplementation appears to be a simple, convenient, and economy intervention to ameliorate these adverse effects. The clinical rational application of vitamin D3 requires a deeper understanding of its mechanisms of action in follicular development. Vitamin D3 is locally synthesized in follicles, and VDRs are widely distributed within follicles, indicating a close relationship between vitamin D3 and follicles. Multiple lines of evidence suggest that vitamin D3 has a potential role in the transition from primordial follicle activation to dominant follicle formation and subsequent development and maturation. It also promotes GCs proliferation and follicle growth in the later stages of follicular development. This review summarizes the effects of vitamin D3 on follicular development and its potential mechanisms. Vitamin D3 promotes follicular development by regulating molecules involved in oocyte meiosis and the secretion of steroid hormones by GCs. On one hand, vitamin D3 can mitigate the adverse effects of excessive testosterone on gap junction communication between GCs, support cyclic guanosine monophosphate (cGMP) transportation, and promotes recovery from oocyte meiotic arrest. On the other hand, vitamin D3 can reduce the expression of TGF-β1in GCs, thereby inhibiting TGF-β1-mediated oocyte meiotic arrest and promoting oocyte development. Additionally, vitamin D3 promotes GCs proliferation by regulating the expression of cell cycle-related genes. Furthermore, vitamin D3 has been shown to have antioxidant or anti-apoptotic effects to protect follicles. This not only benefits the rational use of vitamin D3 to improve various diseases of the female reproductive system, such as improving ovulation in PCOS patients but also improves assisted reproductive technologies. Applying vitamin D3 to the culture of oocytes before and after cryopreservation will increase the in vitro maturation rate of oocytes, providing convenience for the in vitro development of assisted reproductive technologies. However, due to the difficulties in the in vitro preservation and culture of early follicles, there is a lack of exploration into the mechanism of vitamin D3 in early follicle development. Therefore, more experiments are needed to investigate the detailed pathways of vitamin D3 in the development of oocytes in early follicles and follicle maturation. Additionally, the overall impact of vitamin D3 supplementation on ovarian function in vivo is related to the baseline levels of vitamin D in patients, as well as the form, dosage, and timing of vitamin D administration. Thus, further randomized controlled studies are still needed to explore these issues.

### Electronic supplementary material

Below is the link to the electronic supplementary material.

Supplementary Material 1

Supplementary Material 2

Supplementary Material 3

## Data Availability

No datasets were generated or analysed during the current study.
