# Supplementary figures and images for: The role of vitamin D3 in follicle development
Source: J Ovarian Res. 2024 Jul 17;17:148. doi: 10.1186/s13048-024-01454-9 (PMC11253454; doi:10.1186/s13048-024-01454-9)

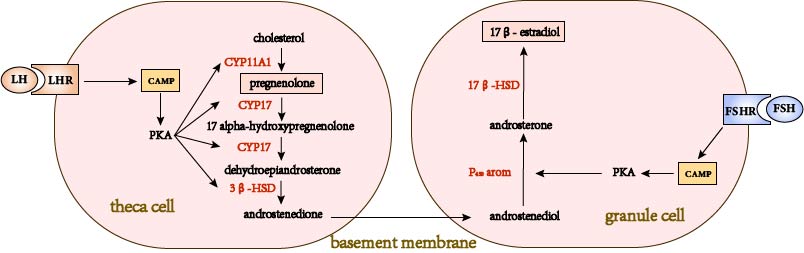

Supplement: Supplementary file 3 — Supplementary Material 3 [file 13048_2024_1454_MOESM3_ESM.jpg]
